# Multidisciplinary protocol and outcomes in placenta accreta spectrum: a 12 year cohort study

**DOI:** 10.1007/s00404-025-08263-5

**Published:** 2026-02-06

**Authors:** Ari Luder, Elias Castel, Nir Kleinmann, Shalom Mazaki-Tovi, Hila Lahav-Ezea, Asaf Shvero, Dorit E. Zilberman, Zohar A. Dotan

**Affiliations:** 1 Department of Urology, Sheba Medical Center, Tel Hashomer, Ramat Gan, Israel; 2 Department of Obstetrics and Gynecology, Sheba Medical Center, Tel Hashomer, Ramat Gan, Israel; 3 Affiliated with the Sackler School of Medicine, Tel Aviv University, Tel Aviv-Yafo, Israel

**Keywords:** Placenta accreta spectrum, Multidisciplinary team, Ureteral catheterization, Maternal morbidity, Cesarean delivery

## Abstract

**Background:**

Placenta accreta spectrum (PAS) is a high-risk obstetric condition associated with hemorrhage, urologic injury, and peripartum hysterectomy. Rising cesarean delivery rates continue to increase its prevalence. Variation in surgical management and limited multidisciplinary involvement may contribute to adverse maternal outcomes.

**Objective:**

To evaluate whether the implementation of a multidisciplinary team (MDT) protocol for PAS was associated with improved perioperative outcomes.

**Study design:**

This retrospective cohort study included 417 women diagnosed with PAS from 2011 to 2022 at a tertiary center. In 2019, a structured MDT protocol was adopted, incorporating standardized imaging, preoperative conference, routine bilateral ureteral catheter (UC) placement, and on-site urologic support. Outcomes of MDT-managed patients (*n* = 108) were compared with pre-MDT patients (*n* = 309). Multivariable logistic regression and generalized linear models adjusted for maternal age, gravidity, prior cesarean delivery, placenta previa, PAS grade, surgical urgency, gestational age, and year of delivery.

**Results:**

After adjustment, MDT care was associated with lower odds of urologic injury (aOR 0.34; 95% CI 0.12–0.82), surgical complications (aOR 0.39; 95% CI 0.18–0.78), transfusion (aOR 0.41; 95% CI 0.14–0.93), and hysterectomy (aOR 0.22; 95% CI 0.05–0.91). Adjusted estimated blood loss decreased by 260 mL (95% CI − 480 to − 70), and length of stay was reduced by 0.9 days (95% CI − 1.4 to − 0.3). Results remained consistent in sensitivity analyses limited to 2017–2022.

**Conclusion:**

Implementation of an MDT protocol was associated with reduced perioperative morbidity, supporting multidisciplinary management as a potentially safer strategy for high-risk PAS surgery.

## What does this study add

**Table:** 

This 12-year cohort provides adjusted evidence that an MDT protocol—including standardized imaging, preoperative joint planning, and routine ureteral catheterization—is associated with improved perioperative safety in PAS surgery.	

## Introduction

Placenta accreta spectrum (PAS) represents a major cause of severe maternal morbidity, including massive hemorrhage, urologic injury, and peripartum hysterectomy. Its incidence has risen substantially in parallel with global increases in cesarean delivery (CD) rates. Early reports estimated PAS prevalence at 1 in 2500–4000 deliveries, increasing to 1 in 533 by the early 2000s and to 1 in 313 in contemporary U.S. datasets [1, 2].

Prior cesarean delivery is the strongest risk factor for PAS, with risk rising progressively with each additional CD [3]. Placenta previa is an especially critical risk factor, conferring markedly increased rates of hemorrhage, hysterectomy, and surgical morbidity [5, 6]. Additional contributors include advanced maternal age, assisted reproduction, and prior uterine surgery [7].

PAS surgery poses a substantial risk of urologic complications due to distorted pelvic anatomy and bladder infiltration, with a reported pooled incidence of ~ 15% in systematic reviews [8]. Complex cesarean deliveries, including those with previa or abnormal adherence, are also associated with elevated risks of relaparotomy and repeat surgery [9].

In 2019, FIGO introduced standardized intraoperative grading (Grades 1–3) to improve diagnostic and operative consistency [10]. Earlier cases must often be re-graded retrospectively for comparability.

Multidisciplinary team (MDT) management, including obstetrics, urology, anesthesia, radiology, neonatology, and specialized nursing has been increasingly advocated. Previous reports demonstrate benefits such as reduced blood loss and improved intraoperative coordination [11, 12]; however, evidence regarding the reduction in urologic injury remains inconsistent due to limited sample sizes and incomplete adjustment for confounders.

In January 2019, our center implemented a structured MDT protocol. Given the 12 year span of evolving imaging and surgical experience, it is essential to distinguish MDT-related improvements from secular trends. This study evaluates whether the MDT protocol was associated with improved perioperative outcomes after adjusting for case mix, urgency, PAS grade, and year of delivery.

## Materials and methods

### Study design and setting

We conducted a retrospective cohort study at Sheba medical center, a tertiary referral center with a dedicated PAS program. All women diagnosed with placenta accreta spectrum (PAS) who underwent delivery between January 1, 2011, and December 31, 2022, were included. In January 2019, a structured multidisciplinary team (MDT) protocol was implemented; therefore, the cohort was divided into two groups:

Pre-MDT (2011–2018) and MDT (2019–2022).

**Fig. 1:**
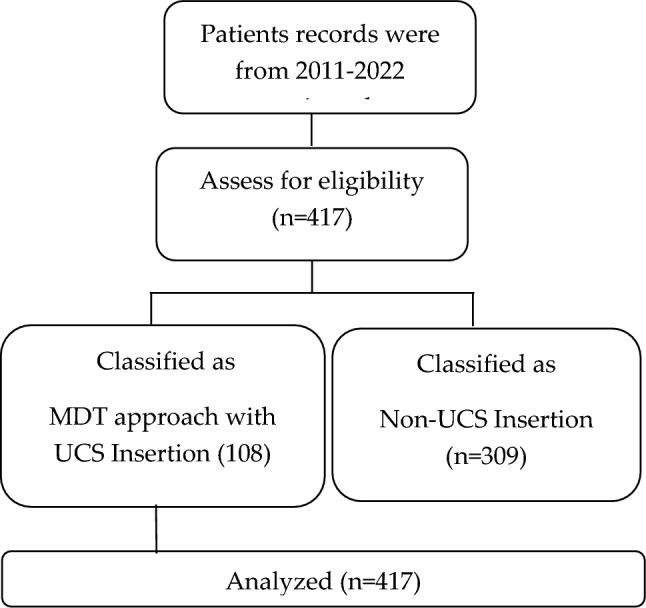
STROBE flow diagram of case identification, eligibility assessment, and cohort allocation. Flow diagram showing the selection of PAS cases. Of 437 records screened (2011–2022), 20 were excluded due to incomplete or incorrect data, leaving 417 eligible patients. These were allocated to the MDT group (*n* = 108) or the non-MDT group (*n* = 309), with all cases included in the final analysis

A STROBE-compliant flow diagram summarizing case identification, exclusions, and final cohort allocation was generated (Fig. 1).

### Case identification

PAS cases were identified using a combination of ICD-9 diagnostic codes, operative reports, and pathology reports when available.

PAS Grading and Retrospective Re-assessment because standardized intraoperative grading was introduced only in 2019, all pre-2019 cases were retrospectively re-graded according to FIGO 2019 criteria (Grades 1–3).

Two senior obstetricians independently reviewed each case blinded to MDT status and outcomes, and discrepancies were resolved by consensus.

Inter-rater agreement could not be calculated because double readings were unavailable for some historical cases exposure.

Definition: MDT protocol.

The MDT protocol included five core components: standardized PAS-targeted ultrasound, including mapping of placental invasion and bladder–uterine interface.

Preoperative MDT conference, involving obstetrics, urology, anesthesia, and radiology.

Routine bilateral ureteral catheter (UC) placement before cesarean delivery.

Dedicated PAS-trained surgical team, experienced in conservative techniques and hysterectomy.

Immediate availability of an on-site urologist throughout the procedure.

Adherence to each component was prospectively documented and is reported in Supplementary Table S1.

### Outcome definitions

Primary outcome urologic injury, including cystotomy, partial or complete ureteral injury, and injuries diagnosed intraoperatively or within 30 postoperative days.

Secondary outcomes any surgical complication, including intestinal injury, uterine rupture, ileus, sepsis, relaparotomy, or other major intraoperative events.

Transfusion, defined as administration of ≥ 1 unit of packed red blood cells. Transfusion decisions followed institutional guidelines.

Intraoperative hysterectomy, Intensive care unit (ICU) admission, Re-operation within 30 days.

Estimated blood loss (EBL), determined by combined suction–irrigation volume and gravimetric sponge assessment, length of stay (LOS), measured in total hospital days; discharge timing was not influenced by fast-track protocols.

### Ureteral catheter–specific outcomes

We recorded: Hematuria, Catheter-associated urinary tract infection (UTI) Acute kidney injury attributable to UC.

### Duration until UC removal

UC-specific outcome data were available for ≥ 90% of MDT cases; missing data were handled as described below. If any UC-related variables lacked documentation, this limitation is acknowledged in the Discussion.

### Covariates

Models were adjusted for the following clinically relevant variables: maternal age, gravidity and parity, prior cesarean deliveries, placenta previa, PAS grade (FIGO 2019: Grades 1–3).

Gestational age at delivery, surgical strategy (conservative management vs cesarean hysterectomy), Planned vs emergent surgery, to adjust for secular trends, evolving practice, and institutional learning effects.

### Statistical analysis

Binary outcomes were evaluated using multivariable logistic regression.

Continuous outcomes were analyzed using: generalized linear models with gamma distribution for EBL Linear regression with robust standard errors for LOS.

### Variable selection criteria

Variables were chosen based on: clinical relevance, prior literature, > 10% change-in-estimate criteria.

### Mediation analysis

To explore whether ureteral catheter use contributed to observed associations, analyses were repeated including UC placement as a covariate.

### Sensitivity analyses

Two sensitivity analyses were performed: Restriction to cases from 2017–2022 to minimize early-era learning curve effects.

Models excluding UC placement to assess robustness of the MDT effect.

### Missing data

Missingness was < 3% for all variables.

A complete-case analysis was performed.

Variable-specific missingness is presented in Supplementary Table S1.

### Software

Analyses were conducted using:

SPSS v23 (IBM Corp.)

Python statsmodels v0.14.

### Ethics and data availability

This study was approved by the Institutional Review Board of Sheba Medical Center (SMC 9269–22). Informed consent was waived due to the retrospective nature of the study. Data are available from the corresponding author upon reasonable request.

## Results

### Cohort characteristics

A total of 417 women with placenta accreta spectrum (PAS) were included in the analysis (108 managed under the MDT protocol and 309 under non-MDT care). Baseline demographic and clinical characteristics are presented in Table 1.

**Table 1:** Demographics and clinical characteristics of the placenta accreta spectrum patients

Variables	Total (*n* = 417)	Study group		*P* value
		MDT Approach (*n* = 108)	Non-MDT approach (*n* = 309)	
Age > 35, years	417 (100)	37.0 (5.04)	36.3 (5.11)	.136
Gravidity	417 (100)	4.1 (1.5)	4.9 (1.5)	.001
Length of gestation, weeks	417 (100)	37 ± 2 (0.875)	36 ± 2 (3.183)	.044
Previous cesarean section, *n* (%)	355 (85.1)	108 (100.0)	247 (79.9)	.001
Accreta degree, *n* (%)				
Placenta accreta	113(27.1)	28 (25.61)	85 (27.74)	.523
Placenta increta	78 (18.9)	20 (19.3)	58 (19.0)	
Placenta percreta	226 (54.2)	59 (55.1)	167 (54.1)	
Placenta previa, *n* %	216 (51.8)	61 (56.5)	155 (50.2)	.330

Several clinically relevant differences were observed between groups. The non-MDT cohort had higher gravidity, whereas the MDT group had a higher proportion of prior cesarean deliveries (100% vs. 79.9%). Gestational age at delivery was slightly higher in the MDT cohort. To enhance completeness and alignment with reporting standards, Table 1 has been revised to include body mass index (BMI), parity, placenta location, prior uterine surgery, in vitro fertilization (IVF), relevant comorbidities (hypertensive disorders and diabetes), placenta previa subtype, planned versus emergent surgery, and surgical strategy (conservative management vs. hysterectomy).

### Unadjusted outcomes

Table 2 has been expanded to include additional clinically important outcomes: transfusion, intensive care unit (ICU) admission, re-operation within 30 days, and neonatal outcomes. Unadjusted analyses demonstrated lower rates of urologic injury, surgical complications, transfusion, and hysterectomy in the MDT group.

**Table 2:** Length of stay, estimated blood loss, and urological and overall complication rate among the multidisciplinary team (MDT) protocol group and the non-MDT (interdisciplinary) group

Variables	Total (*n* = 417)	Study group		*P* value
		MDT approach (*n* = 108)	Non-MDT approach (*n* = 309)	
Estimated blood loss, ml	417 (100.0)	1297 (399)	1586 (1332)	.036
Urgent hysterectomy rate (%)	24 (5.8%)	1 (0.9%)	23 (7.4%)	.04
Urological complications, *n* (%)	48 ( 11.5)	5 (4.60)	43 (14.0)	.046
Surgical complications, *n* (%)	56 (13.42)	6 (5.6)	50 (15.6)	.027
Length of stay, days	417 (100.0)	4.8	5.7	.007
(ICU) admission, *n* (%)	74 (17.7)	12 (11.1)	62 (20.1)	.032
Re-operation ≤ 30 days, *n* (%	26 (6.2)	4 (3.7)	22 (7.1)	.148
Neonatal NICU admission, *n* (%)	98 (23.50)	12 (11.1)	86 ( 27.83)	.011

Continuous outcomes: EBL: − 260 mL (95% CI − 480 to − 70), LOS: − 0.9 days (95% CI − 1.4 to − 0.3).

Sensitivity analysis (2017–2022) Effect sizes remained similar; direction unchanged.

Component analysis When UC placement was removed from the model, the MDT effect on urologic injury was attenuated (aOR 0.51), suggesting partial mediation (Table 3).

**Table 3:** Adjusted outcomes-association of MDT vs non-MDT with primary outcomes

Outcome	aOR (95% CI)	*P* value
Urologic injury	0.34 (0.12–0.82)	.036
Surgical complications	0.39 (0.18–0.78)	.04
Blood transfusion	0.41 (0.14–0.93)	.046
Hysterectomy	0.22 (0.05–0.91)	.027

## Discussion

In this 12 year cohort of women with placenta accreta spectrum (PAS), implementation of a structured multidisciplinary team (MDT) protocol was associated with improved perioperative outcomes, including lower odds of urologic injury, surgical complications, transfusion, and hysterectomy, along with reduced estimated blood loss and shorter hospitalization. These associations persisted after adjustment for case mix, PAS grade, surgical urgency, and secular trends, suggesting that coordinated multidisciplinary care may contribute to safer operative management for high-risk PAS cases.

Context within the existing literature.

The rising incidence of PAS parallels global increases in cesarean delivery (CD), as demonstrated in large population-based analyses. Prior CD is the dominant risk factor for PAS, with a clear dose–response relationship [1, 3], and placenta previa significantly heightens the risk of hemorrhage, surgical morbidity, and hysterectomy [4, 5]. Consistent with these reports, our cohort included a high prevalence of placenta previa and prior CD, particularly in the MDT era.

Published evidence has consistently emphasized the complexity of PAS surgery. Systematic reviews have reported urologic injuries in up to 15% of cases, mainly cystotomy and ureteral damage, underscoring the challenges of distorted anatomy and bladder invasion [6]. Additionally, difficult cesarean deliveries, especially those involving previa or abnormal adherence, are major contributors to postoperative relaparotomy and severe morbidity [7]. These data highlight the importance of structured preoperative planning and intraoperative preparedness.

Earlier studies examining MDT approaches have reported reductions in blood loss and perioperative morbidity, but most were limited by smaller sample sizes, unadjusted analyses, or limited ability to evaluate urologic outcomes [8–10]. Our findings expand on prior work by incorporating rigorous adjustment for confounding variables—including prior CD, PAS grade, surgical urgency, and year of delivery and providing one of the largest adjusted analyses to date regarding urologic injury in PAS surgery.

Interpretation of MDT components.

Component-level analysis helps elucidate potential mechanisms. Routine ureteral catheter (UC) placement was associated with partial mediation of the MDT effect on urologic injury. This is consistent with previous studies and meta-analyses demonstrating that UC can facilitate intraoperative ureteral identification and early detection of injury, even though it does not eliminate the risk entirely [11, 17]. UC-related complications, while present in other pelvic surgeries, were generally low and manageable in this cohort. The availability of an on-site urologist another core MDT component likely further contributed to the timely recognition and management of urologic injuries.

In addition, standardized PAS-targeted imaging, preoperative multidisciplinary planning, and involvement of a dedicated PAS-trained surgical team provide a coordinated structure that may enhance situational awareness, improve surgical sequencing, and reduce urgent intraoperative decision-making. These elements mirror professional society recommendations advocating multidisciplinary care for severe PAS cases.

Secular trends and confounding.

Given the long study period, the potential for improved outcomes related solely to accumulating institutional experience rather than the MDT protocol required careful evaluation. By incorporating year of delivery as a covariate and performing a sensitivity analysis restricted to 2017–2022, we found no evidence that calendar year alone explained the observed differences. This supports the interpretation that the MDT protocol, rather than secular trends, was associated with improved outcomes.

Importantly, the study also addressed baseline imbalances in gravidity, prior CD rate, and gestational age, all of which are known predictors of PAS severity and surgical morbidity. Rigorous adjustment for these variables strengthens the internal validity of the findings.

Clinical implications.

These results support structured multidisciplinary care, particularly for patients with:

- High-grade PAS (FIGO Grade 3)
- Documented bladder invasion
- Multiple prior cesarean deliveries
- Emergency presentations or distorted pelvic anatomy

For institutions without immediate access to urologic surgeons or dedicated PAS teams, feasible adaptations may include: selective UC use in high-risk cases; standardized PAS imaging protocols; regional referral pathways; intraoperative tele-mentoring; and simulation-based team preparation. Evaluating the resource and cost implications of MDT implementation is an important direction for future work, particularly in lower-volume or resource-limited settings.

Strengths.

- This study has several notable strengths:
- A large single-center cohort spanning more than a decade
- Blinded reclassification of PAS grade using FIGO 2019 criteria
- Comprehensive adjustment for key confounders, including surgical strategy
- Use of year-adjusted models to account for secular trends
- Sensitivity analyses demonstrating robustness across time periods
- Component-level mediation analysis providing mechanistic insight

### Limitations

Several limitations should be acknowledged. First, the retrospective observational design limits causal inference. Second, as a single high-volume tertiary center, generalizability to lower-volume institutions may be limited. Third, routine ureteral catheterization a major MDT component may not be feasible in all settings, and documentation of UC-specific outcomes such as hematuria or catheter-associated UTI was incomplete in a small number of cases, which may have resulted in underestimation of the true incidence of catheter-related complications. Fourth, although multivariable modeling adjusted for many influential factors, unmeasured confounding remains possible. Finally, the rarity of certain outcomes, including ureteral injury and hysterectomy, resulted in wider confidence intervals for some effect estimates.

## Conclusion

In this 12 year cohort, a structured multidisciplinary team protocol for PAS was associated with improved perioperative outcomes, including reduced urologic injury, hemorrhage, transfusion, and surgical morbidity. These findings support multidisciplinary management as a potentially safer approach for high-risk PAS cases and highlight the value of coordinated preoperative planning, intraoperative urologic support, and standardized imaging. Further multicenter studies are needed to validate these findings and define optimal implementation strategies across diverse clinical settings.

### What are the key findings?

Compared with pre-MDT management, MDT care was associated with reduced urologic injury, fewer surgical complications, decreased blood loss, lower transfusion requirements, and shorter hospitalization.

## Data Availability

The datasets used and/or analyzed during the current study are available from the corresponding author on reasonable request.
